# Short-Term Mortality Associated With Hypertensive Emergencies: A Prospective Observational Cohort Study From South India

**DOI:** 10.7759/cureus.44150

**Published:** 2023-08-26

**Authors:** Adlin Lawrence, Soumya Syriac, Soumya Umesh, Deepak Kamath, John Michael Raj A, Thenmozhi Nagarajan

**Affiliations:** 1 Internal Medicine, St. John's Medical College Hospital, Bengaluru, IND; 2 General Medicine, St. John's Medical College Hospital, Bengaluru, IND; 3 Pharmacology, St. John's Medical College Hospital, Bengaluru, IND; 4 Biostatistics, St. John's Medical College Hospital, Bengaluru, IND

**Keywords:** medication compliance, end-organ damage, telephonic interview, outcomes of hypertensive emergency, short-term mortality, hypertensive emergency, systemic hypertension

## Abstract

Background and aims

Hypertensive emergencies are caused by acutely occurring massive elevations in blood pressure with features suggestive of acute end-organ damage and are a common complication of hypertension. About 1-2% of all patients with hypertension develop this complication in their lifetime. This study was undertaken to assess short-term outcomes associated with hypertensive emergencies in a tertiary care center.

Methods

We conducted a prospective cohort study and recruited 66 consenting adults with a hypertensive emergency. Sociodemographic details, clinical characteristics, blood pressure readings at different intervals, in-hospital course, and diagnosis of end-organ damage were recorded. The in-hospital outcome was noted as dead or alive. After four weeks, patients were followed up through telephonic interviews and the patient’s status was then reviewed and recorded. Multiple logistic regression determined the predictors of death. Data were analyzed in SPSS version 26.0 (IBM Corp., Armonk, NY, USA).

Results

A total of 66 patients were enrolled, with a mean age of 54.57 (±38.18) years and a male predominance of 44 (66.35%) patients. The majority of patients were known hypertensives (n=55, 83.35%). Of the known hypertensives, 41 (74.54%) patients had discontinued their anti-hypertensive medications prior to admission. The median duration of hospitalization was 10 (7-14) days. The most common presenting complaints were dyspnea (n=35, 53.03%), pedal edema (n=29, 43.94%) and headache (n=25, 37.87%). Forty-one (62.12%) patients required ICU care, and 39 (59.09%) required ventilator support. The most common end-organ damage was acute-on-chronic kidney disease (n=21, 31.81%). The short-term mortality documented at the end of one month was 24 (36.36%). Of these, seven (10.6%) patients died in the hospital, and 17 (25.75) patients died within one month of getting discharged from the hospital. The factors that were associated with high mortality were newly-diagnosed hypertension and in-hospital hypotension.

Conclusion

We found high mortality associated with hypertensive emergencies. At one month follow-up, we found that more than one-third of the patients had died. Post-hospitalisation mortality was higher than in-hospital mortality. Most patients had discontinued their anti-hypertensive medication before admission. The most frequently encountered end-organ damage was acute-on-chronic kidney disease. The factors associated with high mortality were newly-diagnosed hypertension and in-hospital hypotension.

## Introduction

Hypertension is a significant modifiable risk factor leading to cardiovascular diseases. In 2019, the prevalence of hypertension in India was approximately 30.7% [1]. Hypertensive emergency is an acute complication of hypertension, which has a high impact on cardiovascular health and mortality. Among diagnosed hypertensives, 1-2% develop hypertensive emergencies at least once during their lifetime [2].

Hypertensive emergencies are a heterogeneous group of disorders defined as acute elevations in systolic blood pressure (SBP) > 180 mmHg and/or diastolic blood pressure (DBP) >120 mmHg, with features suggestive of progressive or impending acute end-organ damage. This has often been attributed to inadequate treatment or discontinuation of anti-hypertensive medications [3]. Patients usually present to the emergency department with symptoms such as a headache, dizziness, chest pain, dyspnea, altered mental status or other symptoms suggestive of end-organ damage [4]. Acute end-organ damage may be in the form of myocardial infarction, stroke, acute kidney injury, pulmonary oedema, aortic dissection and hypertensive encephalopathy [5]. These conditions often warrant using intravenous anti-hypertensives to control blood pressure.

Literature has repeatedly highlighted the significant morbidity and mortality associated with such acute elevations in blood pressure. A study conducted among patients with a hypertensive crisis in Vishakhapatnam demonstrated an in-hospital mortality of 14%, i.e., seven out of 50 patients succumbed [6]. Vlcek et al. conducted a study among 384 patients and found that 19% of patients with hypertensive emergency had consequent cardiovascular events [7]. She also observed a higher incidence of cardiovascular events in people with hypertensive emergency compared to hypertensives with similar BP readings. Risk factors associated with ‘hypertensive crisis’ (SBP >200 mmHg and/or DBP >120 mmHg) were found to be obesity, female sex, coronary heart disease and noncompliance with medication [7]. A study conducted to estimate the mortality among patients with hypertensive emergency found that the median survival of patients with neurovascular and cardiovascular end-organ damage was 14 days and 50 days, respectively [8].

The physiological and lifestyle characteristics of the Indian population predispose them to develop hypertension and its complications thereof. While adequate data is available pertaining to the management of chronic hypertension, there is a paucity of data regarding the management and outcomes of hypertensive emergencies in the Indian sub-continent. Therefore, this study was undertaken with the objective of assessing the in-hospital course and estimating the short-term mortality of patients with hypertensive emergencies in a tertiary care center.

## Materials and methods

A prospective observational cohort study of 66 patients was conducted for one year starting from June 2021 for all consenting adults with a hypertensive emergency (systolic blood pressure of >180 mmHg or diastolic blood pressure of >120 mmHg, with evidence of acute end-organ damage, either clinically or based on laboratory reports). Pregnant women were excluded from the study since they form a different heterogenous group with different management measures and outcomes.

Ethical clearance

This study was granted approval by the Institutional Ethics Committee (IEC), St. John's Medical College Hospital (study reference number: 354/2019).

Sample size

The sample size was calculated based on a study done by Euro-STAT, a multicenter European study, which estimated outcomes of hypertensive emergencies in 794 patients [9]. They observed a mortality rate of 4%. Using this as the estimated prevalence of mortality at a relative precision of 5% and at a 95% confidence level, we estimated that the minimum sample size required for our study would be 66 patients with a hypertensive emergency [9].

Data collection instrument

A patient information sheet containing the study rationale, noting strict voluntary participation, was provided. Informed consent was obtained from the patients or legally acceptable representative (LAR) for critically ill patients. Data were collected as per the structured proforma. Various sections of the form were:

Socio-Demographic Characteristics

It mentioned age, gender, socio-economic status, and residence.

Clinical Characteristics

It mentioned a brief history indicating symptoms and duration of hypertension.

Comorbidities

It mentioned diabetes mellitus, ischemic heart disease, chronic kidney disease, chronic liver disease, bronchial asthma and other comorbidities, in addition to hypertension.

Treatment Details

Anti-hypertensive medications that patients were taking and compliance with them.

Blood Pressure Recordings

Blood pressure readings at admission, at one hour, at two-six hours, at 24 hours and at the time of discharge were recorded.

In-hospital Course

The procedures done in the hospital and drugs administered for high BP readings were noted.

Investigations for End-organ Damage

Investigations to detect end-organ damage were carried out: ECG to detect hypertensive or ischemic changes, echocardiography to detect right wall motion abnormalities (RWMA), left ventricular hypertrophy (LVH) and ischemic changes; neuroimaging of the brain to detect infarct/bleeding; ultrasonography (USG) abdomen was also done.

Criteria for specific end-organ damage were as follows:

-Acute kidney injury was defined as an increase in serum creatinine by 0.3 mg/dL or more within 48 hours or an increase in serum creatinine to 1.5 times baseline or more within the last seven days or urine output less than 0.5 mL/kg/h for six hours [10].

-Acute coronary syndrome was defined by electrocardiograph (ECG) changes and elevations in specific serum cardiac markers.

-In-hospital hypotension was defined as either an episode of hypotension requiring discontinuation of the antihypertensive drug and/or the administration of vasopressors or fluids [9].

Medical or Surgical Management Measures

The need for intravenous anti-hypertensive drugs was noted, and the duration of the same. Any medical/surgical intervention done to improve the status of the patient and the requirement of facilities needed to continue the management of the patient were noted.

In-hospital Outcome

The in-hospital outcome was recorded as alive or dead. Patients who were discharged against medical advice (DAMA) or those who opted for no resuscitation (DNR) were also recorded.

Outcome at One Month

After four weeks, patients were followed up through telephonic interviews. The patient’s status was then reviewed and recorded.

Study tools

Proforma-Operational definitions - The patients were classified based on the European Society of Cardiology classification and on the presence of acute hypertension-mediated end-organ damage to the heart, retina, kidney, large arteries and brain [9].

Statistical analysis

The data collected were entered in Microsoft Excel (Microsoft® Corp., Redmond, WA, USA) and analyzed using Statistical Package for the Social Sciences (SPSS) version 26.0 (IBM Corp., Armonk, NY, USA). The socio-demographic variables and clinical variables of the study population were presented using descriptive statistics like mean, standard deviation (SD), median and interquartile range (IQR). The outcome variable in our study was all deaths at four weeks after discharge. Chi-square test of association, Fischer exact test and T-test were used as applicable. All the variables that show significant association for death in bivariate analysis were included in the multiple logistic regression model, with death as the dependent variable and the predictors from clinical and socio-demographic variables as covariates. Multiple logistic regression determined the predictors of death. The results were expressed as odds ratio with confidence interval and adjusted odds ratio for multivariate analysis. A p-value of <0.05 was considered significant for all analyses.

## Results

We found that, out of 66 patients hospitalized with a hypertensive emergency, 24 (36.36%) patients died within one month of hospitalization. Of these, seven (10.6%) patients died in the hospital, and 17 (25.75%) patients died within one month of getting discharged from the hospital.

The socio-demographic and clinical characteristics of the patients are depicted in Table 1. A total of 66 patients were enrolled, with a mean age of 54.57 (± 38.18) years and a male predominance of 44 (66.35%) patients. Most patients were known hypertensives (n=55, 83.35%), while only 11 (16.65%) patients were newly diagnosed with hypertension. The predominant comorbidities, in addition to hypertension, were chronic kidney disease (n=37, 56%), hypertensive retinopathy (n=27, 40%) and diabetes (n=25, 37.8%). While most of the patients were on treatment with calcium channel blockers (CCB) (n=50, 90.9%), alpha-blockers and beta blockers were used in 15 (27%) and eight (14.5%) patients, respectively, to control BP. Among the known hypertensives, 46 (85.1%) patients were receiving their anti-hypertensive drugs as fixed drug combinations. It was also observed that 41 (74.54%) patients had discontinued their anti-hypertensive medication prior to admission. In-hospital deaths were seven (10.6%). The short-term outcome was assessed at the end of one month after discharge, wherein an additional 17 (25.75%) patients were found to have succumbed to illness, and one (1.52%) patient was lost to follow-up.

**Table 1:** Socio-demographic and Clinical Characteristics of Patients * Mean (± SD)

		n (%)
Age in years		54.57 (± 38.18)*
Gender	Male	44 (66.35)
	Female	22 (33.65)
Urban residents		42 (63.65)
Rural residents		24 (36.35)
Newly diagnosed hypertensive patients		11 (16.65)
Known hypertensive patients		55 (83.35)
Comorbidities		
	Chronic kidney disease	37 (56)
	Hypertensive retinopathy	27 (40)
	Diabetes	25 (37.8)
	Ischemic heart disease	9 (13.6)
	Cerebrovascular accident (CVA)	7 (10.6)
	Renal artery stenosis	1 (1.52)
	Heart failure	1 (1.52)
Regular medication	Calcium channel blocker (CCB)	50 (90.9)
	Alpha blocker	15 (27)
	Beta-blocker	8 (14.5)
	Vasodilator	7 (12.7)
	Diuretics	6 (10.9)
Drug compliance	Fixed drug (n=55)	46 (85.1)
	Medication adherence present (n=55)	14 (25.9)
Outcome at discharge	Alive	59 (89.39)
	In-hospital death	7 (10.6)
	Discharge against medical advice	7 (10.6)
Outcome after one month	Alive	41 (62.12)
	Dead	17 (25.75)
	Untraceable	1 (1.52)

Table 2 depicts the in-hospital course of patients. The median duration of hospitalization was 10 (7-14) days. The most common presenting complaints were dyspnea (n=35, 53.03%), pedal edema (n=29, 43.94%) and headache (n=25, 37.87%). Of 41 (62.12%) patients requiring critical care, 39 (59.09%) patients required ventilator support. Intravenous (IV) anti-hypertensives were used in 59 (89.39%) patients, the most common drug being labetalol administered to 34 (57.62%) patients. Most of the patients received a single dose (n=35, 59.32%). In-hospital hypotension was recorded in six (9.09%) patients. Changes consistent with left ventricular hypertrophy were demonstrated in 54 (81.81%) patients. The most common end-organ damage was acute kidney injury (n=21, 31.81%), intracranial hemorrhage (n=17, 25.75%) and acute coronary syndrome (n=7, 10.60%). Chronic kidney disease was the most common chronic complication of hypertension.

**Table 2:** In-hospital Course * median (interquartile range)

		n (%)
Days in hospital		10 (7-14)*
Intravenous anti-hypertensive drug used	Labetalol	34 (57.62)
	Nitroglycerin	17 (28.83)
	Nimodipine	8 (13.55)
Duration of intravenous anti-hypertensive drug	Stat	35 (59.32)
	Infusion	24 (40.67)
Hypotension in hospital		6 (9.09)
Management in hospital	Dialysis	31 (46)
	Statins	29 (43)
	Antiplatelets	24 (36)
	Anticoagulants	24 (36)
	Craniotomy	8 (12)
	Aneurysmal clipping	6 (9)
	Angioplasty	5 (7.5)
European Society of Cardiology (ESC) classification of end-organ damage	Acute-on-chronic kidney disease	34 (52.7)
	Intracranial hemorrhage	25 (37.87)
	Acute coronary syndrome	9 (13.6)
	Heart failure	6 (9.09)
	Acute kidney injury	6 (9.09)
	Acute ischemic stroke	6 (9.09)
	Heart failure, acute-on-chronic kidney disease	3 (4.5)
Discharge medication	Calcium channel blocker	57 (91)
	Beta-blocker	39 (66)
	Alpha-blocker	34 (57)
	Vasodilator	30 (50)
	Central agonist	15 (25)

Table 3 depicts the blood pressure readings at different intervals during hospitalization. Blood pressure was recorded at admission, at one hour, at two-six hours, at 24 hours and at the time of discharge.

**Table 3:** Blood Pressure Readings

Blood pressure (mmHg)	At admission	At one hour	At two-six hours	At 24 hours	At discharge
Systolic blood pressure	205	185	164	147	130
Diastolic blood pressure	121	106	94	86	82
Mean arterial pressure	148	132	117	106	59

Table 4 depicts the univariate analysis of the factors associated with mortality in a hypertensive emergency. The factors that were significantly associated with mortality were newly diagnosed hypertension and in-hospital hypotension.

**Table 4:** Univariate and Multivariate Analysis ESC: European Society of Cardiology

Parameters	Outcome		Odds Ratio	P-Value
	Alive	Dead		
Age	53.71±13.79	56.08±17.34	1.01 (0.97-1.04)	0.620
Gender				
Male	28 (64%)	16 (36%)	1.00 (0.34-2.89)	0.999
Female	14 (64%)	8 (36%)	Reference	
Days in Hospital	10 (7-18)	10 (7.5-11)	0.95 (0.87-1.04)	0.306
Newly diagnosed hypertension				
No	38 (69%)	17 (31%)	Reference	
Yes	4 (36%)	7 (64%)	3.91 (1.00-15.16)	0.049
Echocardiography (ECHO)				
Left ventricular hypertrophy (LVH)	34 (62%)	20 (37%)	0.78 (0.15-3.86)	0.765
Left ventricular global hypokinesia	4 (80%)	1 (20%)	0.33 (0.02-4.7)	0.417
Regional wall motion abnormalities (RWMA)	4 (57%)	3 (43%)	Reference	
Hypotension in hospital				
No	41 (68%)	19 (32%)	Reference	
Yes	1 (17%)	5 (83%)	10.78 (1.17-98.83)	0.035
Papilledema				
No	29 (63%)	17 (37%)	Reference	
Yes	13 (65%)	7 (35%)	0.91	0.879 (0.30-1.06)
Ventilatory support				
No	22 (69%)	10 (31%)	1.51	0.403 (0.55-4.23)
Yes	20 (2%)	14 (1%)	Reference	
Dialysis				
No	23 (66%)	12 (34%)	1.21	0.709 (0.44-3.30)
Yes	19 (61%)	12 (39%)	Reference	
ESC Classification				
Renal failure	18 (67%)	9 (33%)	0.60	0.484 (0.14-2.5)
Acute coronary syndrome	6 (55%)	5 (45%)	Reference	
Neurological	18 (64%)	10 (36%)	0.83	0.763 (0.25-2.7)
Ventilator support				
Invasive	22 (69%)	10 (31%)	2.4	0.12 (0.79-7.2)
Noninvasive	11 (46%)	12 (50%)	0.99	

## Discussion

To the best of our knowledge, this is the first study conducted in South India to assess the short-term mortality associated with hypertensive emergencies. We found high short-term mortality (n=24, 36.36%) among patients admitted with a hypertensive emergency. The in-hospital mortality was seven (10.60%). This is at par with existing literature [11]. However, more than double the number of patients succumbed within a month of getting discharged (n=17, 25.75%). This finding demonstrates how cross-sectional studies underestimate mortality in the setting of hypertensive emergency. The high post-hospitalization mortality clearly points towards the importance of regular follow-up and robust management of high blood pressure at subsequent visits and its complications thereof. The danger associated with a hypertensive emergency does not end with in-hospital management, and the consequences of hypertensive emergencies are more catastrophic than what has been documented in the literature.

Our study found that the mean age of patients affected by hypertensive emergency was 54.57 (±38.18) years and demonstrated a male predilection, with 44 (66.66%) male patients. These findings are consistent with a study from Vishakhapatnam [6]. Sabbatini and Kararigas have documented the influence of estrogen-altering mechanisms, which regulate the renin-angiotensin-aldosterone system (RAAS), sympathetic nervous system and body mass and confer relative protection to females against such acute elevations in blood pressure [12]. The common presenting complaint was dyspnea, followed by pedal edema and headache. This is consistent with the literature [8,13]. These symptoms are indicative of the underlying acute end-organ damage. Long-term elevations in blood pressure burden the physiology of various organs, finally culminating in organ injury. About 80% of the patients demonstrated changes consistent with left ventricular hypertrophy (LVH) on echocardiogram. These changes are evidence of a chronic underlying hypertensive pathophysiology [14]. It is also significant to note that the majority of the patients had discontinued their anti-hypertensive medication prior to admission. A cross-sectional study by Wallbach et al. demonstrated similar results, wherein a large proportion of patients admitted with a hypertensive emergency were non-adherent or partially adherent to treatment [15]. This reiterates the imminent danger associated with poor treatment compliance. It is crucial to point out that our study was conducted during the peak of the Covid pandemic, which may have posed an additional hardship on the patients to visit a hospital for medication titration and prescription due to the widespread lockdown.

The common end-organ damage encountered was acute on acute kidney injury, intracranial hemorrhage, and acute pulmonary edema. This finding is in accordance with a cohort study wherein renal and neurovascular emergencies were the most prevalent and prominent predictors of short-term mortality [8]. Intravenous anti-hypertensive medications were used in 90% of patients, with most patients receiving a single dose. The most common drug used was labetalol. Furthermore, 60% of the patients required critical care, with half of them requiring ventilator support. This poses a tremendous burden on the already limited resources in a critical care setting for an eminently preventable medical emergency.

The predictors of mortality were newly-diagnosed hypertension and in-hospital hypotension. Patients who were newly diagnosed to have hypertension were found to have greater mortality compared to known hypertensives. Literature had attributed this to the relative protection conferred by chronic hypertension-induced arteriolar hypertrophy to end organs in the setting of chronic blood pressure elevation [16]. In newly diagnosed hypertensives, this physiological response takes time to set in, thereby increasing the susceptibility of organs to damage. Furthermore, newly diagnosed hypertension poses an additional risk of being insufficiently evaluated and inadequately treated.

In-hospital hypotension, in the setting of a hypertensive emergency, has been documented by other studies too [11]. There is evidence that points towards upregulation of the sympathetic nervous system secondary to cardiac remodelling encountered in hypertension [17]. When the cardiovascular system is subjected to sustained excitatory sympathetic regulation, it eventually results in heart failure, which may precipitate hypotension with the passage of time [18]. Moreover, iatrogenic hypotension is a preventable complication. There are existing guidelines on blood pressure reduction in the different subgroups of end-organ damage seen in hypertensive emergencies [19].

Limitations

We have not been able to assess adherence quantitatively as the patients were critically ill. Also, we have not been able to accurately diagnose and differentiate between primary and secondary hypertension, as it was an unfunded study. Multivariate analysis could not be performed due to limitations in sample size.

Future studies

Future studies are required with greater sample sizes to identify the factors associated with death for each subgroup of end-organ damage and to develop guidelines for the strategic reduction of blood pressure for respective subgroups. This will help reduce mortality associated with this medical emergency.

Implication

Our study has exhaustive implications for the short-term outcomes of hypertensive emergencies. There is a dire need for stringent policymaking on awareness regarding hypertension and its acute complications. Clinicians need to take on the pivotal role of early detection and robust management of hypertension, with a particular emphasis on health education about medication adherence. The patients and their caregivers, too, must be motivated to take responsibility for medication adherence. Post-hospitalisation course needs frequent monitoring and appropriate management through regularly scheduled visits or teleconsultation. This collective initiative will help create a supportive ecosystem for the patient, where such medical emergencies can be foreseen and prevented.

## Conclusions

We found high short-term mortality associated with hypertensive emergencies. At one month follow-up, we found that more than one-third of the patients had died. Post-hospitalisation mortality was higher than in-hospital mortality. Most of the patients had discontinued their anti-hypertensive medication prior to admission. The most frequently encountered end-organ damage was acute-on-chronic kidney disease. The majority of patients required critical care and invasive ventilation. The factors associated with high mortality were newly-diagnosed hypertension and in-hospital hypotension.
